# Influence of Diet and Lifestyle on the Development of Gestational Diabetes Mellitus and on Perinatal Results

**DOI:** 10.3390/nu14142954

**Published:** 2022-07-19

**Authors:** Alba Yuste Gómez, Maria del Pilar Ramos Álvarez, José Luis Bartha

**Affiliations:** 1 Department of Chemistry and Biochemistry, Faculty of Pharmacy, CEU San Pablo University, 28925 Madrid, Spain; pramos@ceu.es; 2 Director of the Maternal and Fetal Reseach Group, IdiPaz, Department of Maternal Fetal Medicine, Hospital Universitario La Paz, 28046 Madrid, Spain; jose.bartha@uam.es

**Keywords:** gestational diabetes mellitus, pregnancy, nutrition in pregnancy, glycemic index, glycemic control, nutritional education

## Abstract

GDM is a multifactorial disease, so there is controversy regarding the mechanisms involved in its pathogenesis. We speculate whether lifestyle and eating habits influenced the appearance and pathogenesis of GDM. To explore this issue, the aim of the present study was to analyze maternal diet and lifestyle characteristics in early pregnancy and their influence on the development of GDM. The study included 103 pregnant women who completed a questionnaire on nutritional knowledge, lifestyle and eating habits. Perinatal and biochemical outcomes as well as pregestational lifestyle and eating habits were compared between normoglycemic women and those who developed GDM. The results obtained showed that women who developed GDM had erroneous knowledge regarding nutrition. Consumption of white bread (*p* = 0.018), added sugars (*p* = 0.037), legumes (*p* = 0.025), fish (*p* = 0.014), butter (*p* = 0.010) and the performance of less physical activity (*p* = 0.024) correlated with glucose intolerance in pregnant women. In conclusion, we found a relationship between dietary and lifestyle habits at the beginning of pregnancy and the later diagnosis of GDM.

## 1. Introduction

Gestational diabetes mellitus (GDM) is defined as any degree of hyperglycemia that is first recognized during pregnancy [1,2,3]. However, the International Association of Diabetes and Pregnancy Study Groups (IADPSG), World Health Organization (WHO) and the International Federation of Gynecology and Obstetrics (FIGO) have differentiated two categories of hyperglycemia in pregnancy: GDM diagnosed for the first time in pregnancy and overt diabetes, the latter referring to pregestational diabetes or diabetes diagnosed at the first prenatal visit and meeting the criteria for diabetes outside of pregnancy [2,4]. The worldwide prevalence of GDM is 16.5% [1], and according to the International Diabetes Federation (IDF), most cases of hyperglycemia in pregnancy (75%–90%) are GDM. The prevalence varies depending on the criteria used for diagnosis, for which there has been a lack of consensus. Currently, the ADA, the WHO, the FIGO and the Endocrine Society recommend the diagnosis of GDM using the IADPSG criteria [1], which were developed based on the results of the HAPO study [5]. However, the surprisingly high rate of this condition (nearly 30% of all pregnant women) when these criteria are applied in some countries [6] with previously lower prevalence makes one consider whether overdiagnosis stigmatizes women with otherwise problem-free pregnancies. Accordingly, many countries have not accepted these criteria, and some organizations, such as ADA, have reconsidered the continued use of IADPSG in favor of the previous criteria [6,7]. Despite increasing rates of diagnosis and intensive surveillance, hyperglycemia during pregnancy is still associated with short- and long-term health complications for the mother, fetus and newborn. Women with GDM have a higher risk of hypertensive disorders (with preeclampsia being the highest risk) [8,9], vaginal and urinary infections [10], premature birth [5], hydramnios [11] and surgical delivery, in addition to increased risk of developing DM2 and cardiovascular diseases in the long term [1]. Regarding complications in the fetus and/or newborn, macrosomia [2,5] and perinatal hypoglycemia should be highlighted, as they can damage the vital organs of the newborn, such as the brain, and in the long term, cause an increase in the probability of the mother developing DM2 and CVD [1]. It seems that our focus should shift from overdiagnosis to prevention of hyperglycemia in pregnancy, regardless of the cutoffs that can be used as criteria for definitions. Current evidence, as demonstrated in this meta-analysis [12], focuses on whether diet (as a treatment) decreases the complications derived from GDM, but does not show the influence of diet as a method of preventing GDM.

GDM is a disease of multifactorial etiology, so there is controversy regarding the mechanisms involved in its pathogenesis. We speculate whether lifestyle and dietary habits influence the onset and pathogenesis of GDM. One of the environmental factors that most influence GDM is maternal nutrition, given the nutritional dependence of the fetus on the mother, whereby poor or inadequate nutrition of the mother negatively affects fetal development and growth [10]. Certain bacteria that constitute our microbiota are related to insulin resistance [13,14]. This may explain some dietary risk factors for GDM [15,16]. In addition, a deficit of selenium and zinc [17] and an imbalance of B vitamins [18,19,20] can influence the development of GDM. Low vitamin C levels were also associated with an increased risk of GDM [21], as well as vitamin D deficiency [22,23].

Currently, diet and lifestyle are used as tools in the initial treatment of GDM, but are not considered as possible risk factors that influence its development. This approach deserves careful consideration. It is known that the adoption of a healthy dietary pattern has positive effects on the prevention and treatment of diabetes, but few studies have analyzed the impact of the interaction between diet and the etiology, pathogenesis and prognosis of GDM. To shed light on this issue, the objective of the present study was to evaluate the influence of maternal diet and lifestyle in early pregnancy on the further development of GDM and on perinatal outcomes, especially those related to GDM-related complications.

## 2. Materials and Methods

This study was designed as a prospective cohort study, and it was carried out at the La Paz University Hospital and approved by the hospital’s bioethics committee.

### 2.1. Study Population

The target population of the study comprised 104 pregnant women, who, having given informed consent, completed a questionnaire on nutritional knowledge, lifestyle and dietary habits, and who underwent pregnancy monitoring, including biochemical, obstetrics and perinatal assessments. The inclusion criteria were: 1. Maternal age greater than 16 years. 2. Signing of the informed consent. 3. Knowledge and understanding of the language. 4. Gestational age less than 16 weeks. The exclusion criteria were: 1. Non-acceptance of informed consent. 2. Repeated vomiting after OGTT and impossibility of performing diagnostic tests. 3. Known fetal anomaly. 4. Pre-gestational diabetes.

### 2.2. Diagnosis of Gestational Diabetes

The diagnosis of GDM was carried out according to the two-step strategy and to the recommendations of the Spanish Group of Diabetes and Pregnancy [6].

### 2.3. Patient Recruitment

The recruitment of pregnant women took place in the antenatal clinic of the health centers dependent on La Paz University Hospital, Madrid. A total of 104 pregnant women were recruited, from whom data was obtained on their lifestyle and dietary habits and on whom pregnancy follow-up was carried out.

### 2.4. Questionnaire

A specific questionnaire was developed to assess the knowledge of pregnant women about nutritional needs during pregnancy, as well as their lifestyle and dietary habits (see Supplementary Materials File S1). The various items of the questionnaire, divided into blocks, are detailed below (1. Identification data. 2. Demographics. 3. Anthropometric data. 4. Clinical history. 5. Eating habits; food table. 6. Lifestyle. 7. Supplement consumption. 8. Knowledge).

In the fifth block of the questionnaire (eating habits), there was a table of foods that specified both the weekly frequency and the daily rations of the food groups (See Appendix Table A1).

### 2.5. Biochemical Analysis and Clinical Parameters Assessed

Maternal parameters were assessed in the second trimester of pregnancy, including the results of screening and diagnostic tests for GDM (fasting glycemia, O’Sullivan test glycemia at one hour, glycemia determined at one hour, two hours and three hours after the glucose overload of 100 g), basal insulin, HbA1c, ferritin, total protein, hemoglobin, total cholesterol and vitamin D.

Among the maternal outcomes of pregnancy, the following parameters were assessed: blood pressure (BP), final weight, total weight gain, gestational age at delivery, route of delivery, intake of supplements, reason for admission and diagnosis at discharge after delivery.

The neonatal results assessed were newborn weight, height, head circumference, weight percentile and Apgar test at one minute and five minutes of life.

### 2.6. Statistical Analysis

Statistical analysis was performed using the Statistical Package for Social Sciences (SPSS) program. A descriptive analysis of all the variables under study was performed with graphical representation in histogram or sector diagram, depending on whether the variable was quantitative or categorical. The normality test of the quantitative variables was also included in order to use parametric or non-parametric tests in future hypothesis tests.

When the number of variables was greater than fifty, the Kolmogorov–Smirnov statistic was applied for the normality test. The level of significance previously established was 95% (*p* < 0.05).

If a quantitative variable was normally distributed in the groups according to the categorical variable (presence of GDM), means were compared by parametric tests (Student’s *t* test); if it did not have a normal distribution, non-parametric tests (Mann–Whitney’s U test) were used. When a variable had a small sample size in both categories, non-parametric tests were used.

For the comparison of the categorical variables, Fisher’s exact test or chi-square test were used, and the data were shown as number and percentage.

Correlation between variables was performed using Spearman’s correlation analysis, since most of the variables followed a non-normal distribution. The results of the questionnaire were correlated with parameters (HbA1c, O’Sullivan result, basal glycemia, OGTT 100 among others).

## 3. Results

A total of 103 pregnant women completed the questionnaire, and dietary and lifestyle data were obtained. Of these, seventy completed pregnancy follow-up at the La Paz University Hospital. Sixty-eight gave birth at the center, and delivery data were also obtained.

Among these women, the development of a total of 10 cases of GDM (10/70; 14.3%) was detected. This corresponds to an incidence of GDM of 14.3%.

### 3.1. Demographics

Of the pregnant women, 68.9% were European, followed by 10.7% from South America. In the case of foreigners, the mean residence time in Spain was 13.4 ± 9.2 (0.5–33) years. Regarding their marital status, 63.1% (65/103) were married, 35.0% (36/103) were single and two were divorced (1.9%). Regarding occupation, 84.5% worked outside home (87/103) while the rest did not have a job (16/103, 15.6%). The level of education with the highest proportion was the degree/bachelor’s degree/diploma, being 49.9%.

### 3.2. Medical and Obstetric History

Of the pregnant women, 24.3%; (25/103) had a chronic disease, with the most prevalent being asthma (3.9%) and hypothyroidism 5.8% …, and 22.2% (22/103) reported having allergies. Regarding diabetes, 32.4% (33/103) stated that they had first-degree relatives with diabetes, and 70% (7/10) of the pregnant women who developed GDM had first-degree relatives with diabetes. Furthermore, 8.2% had had gestational diabetes in previous pregnancies. Of these, 55.6% required insulin for glycemic control and 44.4% did not, and 60% of them affirmed that they controlled their pre- and postprandial blood glucose levels.

The average weight of previous children was 3310.0 ± 720.8 (2000–6000) g. Regarding previous births, 86.8% of pregnant women had had a vaginal birth in the previous pregnancy and 13.5% had had a previous cesarean section.

### 3.3. Anthropometrics and Body Perception

Pre-pregnancy weight was 63.1 ± 11.0 (42–112) kg, and pre-pregnancy BMI was 23.7 ± 3.9 (15.4–37.2). Regarding their body perception, 82.3% of pregnant women with a BMI of normal weight reported seeing themselves as normal weight, highlighting that only 12.5% of pregnant women with a BMI of obesity considered themselves to be obese.

The results of the comparison of some data (anthropometric, demographic, obstetric history, relatives with Diabetes mellitus) between the two groups (women with GDM and normoglycemic women) are shown in (See Appendix Table A2 and Table A3).

### 3.4. Dietary Habits and Lifestyle

Of the pregnant women, 59.2% reported having a greater appetite since they became pregnant, and 41.2% referred to having cravings. Regarding the number of daily meals, the largest proportion, 39.2% of the pregnant women, ate five meals a day, with only 12.4% having a second dinner. In the food table, the weekly frequency and the daily ration of consumption of each food were specified. (See Appendix Table A1). Concerning physical activity and exercise, 38.2% of the respondents performed physical activity prior to pregnancy, and 31.4% of the women reported being physically active during pregnancy. Thus, physical activity decreased by 7% during pregnancy. In addition, 81% of the women took supplements during pregnancy, while only 49.0% took them in the months before pregnancy.

### 3.5. Consumption of Tobacco, Alcohol, Caffeine and Processed Foods

Of the pregnant women, 20.4% were smokers in the pre-gestational stage, with a consumption of 5.2 ± 4.2 (0–12) of cigarettes per day, and 78.5% of them quit tobacco at the time of pregnancy. Only 1% of the women reported alcohol consumption during pregnancy, while 55.3% consumed caffeine during pregnancy, with a weekly frequency of 5.7 ± 2.3 cups (1–7) per day. Finally, 72.1% of the pregnant women consumed processed foods during pregnancy, with a weekly frequency of consumption being 4.5 ± 2.2 (1–7) days/week.

### 3.6. Biochemical Results

The biochemical evaluation results are shown in (See Appendix Table A4).

### 3.7. Perinatal Results

The perinatal results evaluated are shown in the Appendix A section (See Appendix Table A5). Regarding the route of delivery, of the 103 women recruited, 46.6% of deliveries were spontaneous vaginal deliveries, 3.9% were spontaneous abortions, and 20.3% were cesarean sections.

### 3.8. Comparison of Biochemical and Perinatal Results between the Group of Pregnant Women Who Developed GDM and the Normoglycemic Women

#### 3.8.1. Biochemical Results

Maternal biochemical parameters were studied in the second trimester of pregnancy. A comparison of means was made between the biochemical parameters of the pregnant women who developed GDM and the normoglycemic women (See Table 1). There were no statistically significant differences in the biochemical parameters between the two groups except for the GDM diagnostic tests (basal O’Sullivan, glucose overload of 100 g).

#### 3.8.2. Obstetric and Perinatal Results

Only two cases of pregnancy-induced hypertension (2/68; 2.9%) were detected out of 68 deliveries that took place in the hospital. Further, a total of eight cases of anemia (8/68; 11.8%) were detected. Concerning the perinatal assessment results (final maternal weight, gestational age at delivery, newborn weight and height, head circumference, Apgar test at one minute and at five minutes, percentile weight and total weight gain), no significant differences were obtained between the group of women who developed GDM and those with normoglycemia, with the exception that the gestational age at delivery was lower in pregnant women who developed GDM (See Table 2). In addition, when performing the Spearman’s correlation, there was a negative correlation between gestational age at delivery, fasting glycemia and results from the glucose overloads of 100 g (See Table 3), so it could be said that the higher glucose intolerance the lowered gestational age at delivery.

### 3.9. Relationship between Lifestyle, Dietary Habits and Nutritional Knowledge with the Development of GDM

A summary of food consumption is presented in Table 4, showing weekly frequency of consumption in each study group (pregnant women with GDM and normoglycemic). We found significant differences in the consumption of white bread among pregnant women who develop GDM and controls (See Table 4). Regarding the time of activity during pregnancy (represented in minutes per week), it was lower among those who developed GDM when compared to normoglycemic women, but the differences did not reach statistical significance (See Appendix Table A6).

### 3.10. Relationship between Lifestyle and Eating Habits and Glucose Intolerance

Then we analyzed the relationship (obtained by means of Spearman’s correlation) between certain food groups and physical activity and glucose tolerance tests (see Appendix Table A7, Table A8, Table A9, Table A10, Table A11 and Table A12), to ascertain whether they could be associated with the degree of glucose intolerance in the women that were diagnosed with GDM.

As shown in the Appendix A section, in the OGTT of 100 g glucose, we found a significant negative correlation between physical activity and glycemia at two hours (See Appendix Table A5).

A positive correlation was also observed between a higher consumption of white bread (*p* = 0.018 in OGTT with determination at two hours) and added sugar in beverages and foods with glucose intolerance in OGTT (*p* = 0.037 with fasting glycemia; *p* = 0.009 with glycemia at one hour in the OGTT; *p* = 0.013 with two hours glucose in OGTT; and *p* = 0.021 with glucose at three hours in the OGTT) (See Appendix Table A6 and Table A7). Likewise, there was a negative correlation between a lower consumption of legumes both in weekly frequency and in daily ration with increased fasting glucose (*p* = 0.025 and 0.005) (See Appendix Table A8). We also found a negative correlation between fish consumption (daily ration) and fasting glucose (*p* = 0.014) (See Appendix Table A9). By contrast, the consumption of butter, both in weekly frequency and in daily ration, correlated positively with fasting glycemia (*p* = 0.010, 0.008) (See Appendix Table A10).

### 3.11. Results of the Nutritional Survey Significant in the Development of GDM

Statistical results indicated a borderline confirmation (*p* = 0.05) between the presence of allergies and the development of GDM. Pregnant women who developed GDM had a higher percentage of allergies (40%) compared to normoglycemic women (14.3%). Family history of diabetes was more frequent in women who developed GDM versus normoglycemic patients (80% and 40.7% for GDM and normoglycemic women, respectively; *p* = 0.02).

Regarding the question raised in the questionnaire on the knowledge of pregnant women about whether excessive weight gain during pregnancy can affect the health of the newborn, we found a significant difference between both groups, since 50% of the pregnant women who developed GDM responded that they were not aware of it, compared to 20% of normoglycemic women (*p* = 0.04).

In terms of the number of meals eaten per day, the study showed that it was significantly lower in the GDM group, as only 40% of pregnant women who developed GDM ate snacks compared to 73.3% of normoglycemic women (*p* = 0.036).

Regarding the knowledge of the pregnant women on whether it was correct to consume extra virgin olive oil only in moderation to prevent excess fat/cholesterol in the blood (the correct answer was NO), the result obtained was of borderline significance (*p* = 0.06); 60% of pregnant women who developed GDM believed that they should consume it in moderation, despite its being a healthy fat, compared to 30% of normoglycemic women.

The answer to the question of whether it was correct to consume fruit only in moderation due to its high sugar content (the correct answer being NO) had a borderline significance (*p* = 0.056); we found that that 60% of the pregnant women who developed GDM believed that they should consume it only in moderation, compared to 25% of normoglycemic women. Finally, 30% of pregnant women who developed GDM (*p* = 0.011) considered that their diet and lifestyle were correct, compared to 5% of normoglycemic women.

## 4. Discussion

The objective of this study was to evaluate the influence of maternal diet and lifestyle (before and during the first weeks of gestation) on the development of GDM and on GDM-associated obstetrics and perinatal outcomes. The available evidence focuses on nutrition as a key tool in the initial treatment of GDM, but its possible involvement as a risk factor in the pathogenesis of GDM is not well established [24,25]. The present study demonstrates that an increase in the frequency of consumption of white bread is associated with the development of GDM. White bread is a food with a high glycemic index (GI), so blood glucose levels may be affected, as demonstrated in a meta-analysis showing that low GI diets in pregnant women are associated with better postprandial blood glucose results [12]. Furthermore, the ROLO study showed that women at high risk of developing GDM who had previously given birth to a baby weighing more than 4 kg and who received dietary advice on low GI diets had a lower gestational weight gain, in addition to less glucose intolerance, than those who followed the standard of care [26].

It is evident that there is an association between a decrease in physical activity during pregnancy and the development of GDM. Regular low- or moderate-intensity physical activity reduces both fasting and postprandial blood glucose levels [27]. In addition, it reduces the insulin requirement in pregnant women with GDM [28].

A higher consumption of butter and added sugars leads to greater glucose intolerance, as observed in the GDM diagnostic test (OSB, SOG 100 g). Some previous observational studies show that GDM is more prevalent in women who have a higher intake of meat and a lower intake of whole carbohydrates, fruits, vegetables and fish. Furthermore, a high intake of saturated fats can interfere with insulin signaling, and it also induces inflammation and endothelial dysfunction, both pathogenic factors in GDM [29,30].

The dietary recommendations in pregnancy indicate the realization of six meals a day. In our study, we found a correlation between the absence of snacks and the development of GDM. The group of pregnant women who developed GDM had a higher proportion of women who did not eat snacks, which probably meant that the rest of their meals had a higher glycemic load.

The results of the questionnaire showed that the group of pregnant women who developed GDM had erroneous knowledge about nutrition during gestation. The erroneous conviction that fruits should be consumed only in moderation due to their sugar content could trigger consumption reduction habits, which would imply a decrease in the consumption of micronutrients (vitamins and minerals) essential for pregnancy and the prevention of pathologies. In addition, a lack of knowledge about healthy sugars or carbohydrates could increase the consumption of “no added sugar” products, rich in artificial sweeteners. Previous evidence has shown that certain artificial sweeteners can induce metabolic changes that contribute to the pathophysiological mechanisms of GDM and high neonatal birth weight [31].

In addition, the group of pregnant women who developed GDM responded that they were unaware of the importance of healthy weight gain for the health of the newborn. Moreover, they had the mistaken belief that their lifestyle was correct. These erroneous convictions, together with other risk factors, such as the presence of relatives with diabetes, can influence the development of GDM.

Considering fetal programming and its relevance in the future diseases that children of mothers with GDM may develop, it is essential to improve glucose metabolism and promote the maintenance of a healthy weight. In this scenario, emphasis should be placed on a dietary intake of healthy foods.

This pilot study demonstrates the importance of nutritional education before and during pregnancy, and establishes a basis for future studies on the involvement of diet in the pathophysiology of GDM using a larger cohort, in order to obtain firm conclusions.

## 5. Conclusions

In summary, the present study provides information on the influence of lifestyle and dietary habits on the development of GDM and on perinatal outcomes.

The perinatal and biochemical results obtained in pregnant women with GDM indicate that the glycemic control of the pregnant women was good, but the small number of cases did not allow us to reach definitive conclusions. The results obtained from the nutritional survey showed erroneous knowledge (consumption of white bread, absence of snacks, etc.) about nutrition in the group of women who developed GDM. These misconceptions may have influenced their lifestyle habits, and along with other risk factors, such as having a history of diabetes, increased the risk of developing GDM. In fact, there was an association between the consumption of white bread, added sugar, legumes, fish and butter and the undertaking of less physical activity and the degree of glucose intolerance in pregnant women diagnosed with GDM.

This study demonstrates the importance of nutritional education before and during pregnancy.

Research in large cohorts should be emphasized to identify modifiable risk factors and reduce the prevalence and complications of GDM.

In conclusion, the results obtained showed that women who developed GDM had erroneous knowledge regarding nutrition. Further studies are needed to confirm whether this may definitively influence the development of GDM and its perinatal outcomes.

## Figures and Tables

**Table 1:** Biochemical parameters of the pregnant women with GDM and those with normoglycemia, and statistically significant differences.

Variable	DIABETES												*p*-Value
	NO				YES				Total				
	Mean	SD	Median	Rank	Mean	SD	Median	Rank	Mean	SD	Median	Rank	
Fasting glycemia (mg/dl)	75.5	6.9	74	42	86.5	7.7	82	18	76.7	7.7	75	42	0.001
O’Sullivan 1 h (mg/dl)	118.7	23.2	118	95	172	41.3	179	120	124.7	30.5	120	146	0.002
OGTT basal (mg/dl)	77.8	7.4	76	27	84	8.1	83		80.0	8,1	77	29	0.069
OGTT 1 h (mg/dl)	154.1	26.4	150	70	190.4	19.6	192	73	167.2	29.6	180	99	0.002
OGTT 2 h (mg/dl)	127.3	20.5	127	72	198.4	27.4	186	81	152.9	41.5	149	165	0
OGTT 3 h (mg/dl)	114.1	15.1	115.5	62	167.5	29.3	152	91	133.3	33.4	121	146	0
Fasting insulin (U/mL)					10.2	5.0	9	17	10.2	5.0	9	17	-
HbA1C (%)					5.3	0.3	5.4	1.4	5.3	0.39	5.4	1.4	-
Ferritin (ng/mL)	17.9	17.5	11	84	11,3	6.9	8.5	16	17.2	16.8	10	84	0.466
Total proteins (g/dL)	6.1	0.3	6.2	1.4	6.0	0.2	6	0.8	6.1	0.3	6.2	1.4	0.610
Hemoglobin (g/dL)	11.7	1.0	11.8	5	12,0	1.1	11.9	4.1	11.8	1.0	11.8	5.3	0.452
Total cholesterol (mg/dL)	238.8	35.0	241.5	142	217.9	29.5	218.5	78	233.3	34.6	232	142	0.119
Vitamin D (ng/mL)	21.1	5.8	21.5	20	21		21	0	21.0	5.5	21	20	1

**Table 2:** Perinatal results of pregnant women with GDM and those with normoglycemia.

Variable	DIABETES												*p*-Value
	NO				YES				Total				
	Mean	SD	Median	Rank	Mean	SD	Median	Rank	Mean	SD	Median	Rank	
Final weight (kg)	73.9	9.6	72.3	50.7	73.1	13.8	76.2	40.9	73.8	10.2	73.0	58.2	0.847
GA at delivery (weeks)	38.6	2.5	39	14	37.7	1.0	38	3	38.5	2.4	39	14	0.021
Newborn weight (g)	3163.1	520,9	3120	3490	3133.8	567.3	3340	1655	3159.1	523.0	3122.5	3490	0.918
Newborn height (cm)	48.8	1.9	49	10	48	2.1	48.5	7	48.7	1.9	49	10	0.259
Head circumference (cm)	34.1	1.5	3. 4	9	34,0	1.4	34.5	4.5	34.0	1.5	34.2	9	0.951
Apgar 1	8.6	0.9	9	6	8.8	0.3	9	1	8.6	0.8	9	6	0.411
Apgar 5	9.59	0.8	10	5	9.6	0.5	10	1	9.6	0.8	10	5	0.858
Weight percentile	57.7	25.6	60	96	55.6	37.9	61	90	57.4	27.0	60	96	0.909
Weight gain (Kg)	9.8	4.9	10.5	18.6	10,3	5.1	12.4	13.6	9.8	4.9	10.6	18.6	0.777

**Table 3:** Correlation between gestational age at delivery and diagnostic tests for GDM.

	Rho Spearman	*p*-Value
Gestational age and fasting glycemia	−0.343	0.006
Gestational age and OGTT 1 h	−0.613	0.001
Gestational age and OGTT 2 h	−0.502	0.012
Gestational age and OGTT 3 h	−0.550	0.005

**Table 4:** Food consumption (shown as weekly frequency, fc) of the pregnant women diagnosed with GDM and those without GDM.

Variable	Gestational Diabetes												*p*-Value
	NO				YES				TOTAL				
	Mean	SD	Median	Rank	Mean	SD	Median	Rank	Mean	SD	Median	Rank	
White bread (fc)	5.5	2.2	7	7	6.7	1	7	3	5.6	2.2	7	7	0.012
Cereal (fc)	3.5	1.8	3	6	2.8	2.4	2	7	3.4	1.9	3	7	0.159
Legume (fc)	2.0	1.5	2	7		1.4	2	4	2.1	1.5	2	7	0.599
Vegetable (fc)	4.1	2.3	4	7	4.9	2.6	6.5	6	4.2	2.4	5	7	0.261
Fruit (fc)	5.6	2.2	7	8	5.3	2.8	7	7	5.6	2.3	7	8	0.897
Whole milk (fc)	5.5	2.7	7	7	3.7	3.7	4	7	5.4	2.8	7	7	0.288
Milk 0%	5	3.1	7	7	5.1	2.9	7	7	5.0	3	7	7	0.815
Yogurt (fc)	3.7	2.7	4	7	2.9	3.2	1.5	7	3.6	2.8	4	7	0.393
Red meat (fc)	2.0	1.7	2	7	2.1	2.3	1	7	2.1	1.8	2	7	0.699
White meat (fc)	2.8	1.5	3	7	2.8	1.1	3	4	2.8	1.5	3	7	0.931
Fish/seafood (fc)	1.9	1.3	2	6	1.8	1.2	2	4	1.9	1.3	2	6	0.944
Egg (fc)	2.7	1.5	3	7	3.4	2.7	4	7	2.	1.7	3	7	0.392
Evoo (fc)	5.7	2.5	7	7	6.1	2.2	7	7	5.7	2.4	7	7	0.691
Sunflower oil	1.1	0.9	1	3	1	0	1	0	1.1	0.9	1	3	0.403
Butter (fc)	2.2	2.7	1	7	2.6	2.9	2	7	2.2	2.7	1	7	0.617
Cream (fc)	1.1	1.8	0	7	2.3	2.8	1.5	7	1.2	1.9	0	7	0.295
Dry fruits (fc)	2.0	2.2	2	7		2.7	1.5	7	2.1	2.3	2	7	0.937
Added sugar (fc)	2.9	3.4	0	7	4.2	3.6	7	7	3.1	3.4	0	7	0.277

## Data Availability

Not applicable.

## Supplementary Materials

The following supporting information can be downloaded at: https://www.mdpi.com/article/10.3390/nu14142954/s1, Supplementary Materials File S1: Nutrition Questionnaire.

## Appendix A

**Table A1:** Feeding table.

WEEKLY FREQUENCY/ DAILY CONSUMPTION RATION	N	MINIMUM	MAXIMUM	MEAN	SD
White Bread					
Frequency	100	0	7	5.3	2.3
Ration	100	0	5	1.5	0.8
Wholemeal Bread					
Frequency	4	4	7	6.2	1.5
Ration	4	2	2	2.0	0.0
Cereals					
Frequency	102	0	7	3.5	2.0
Ration	101	0	5	1.2	0.6
Legume					
Frequency	100	0	7	2.1	1.7
Ration	101	0	3	0.9	0.5
Vegetable					
Frequency	101	0	7	4.1	2.4
Ration	101	0	7	1.1	0.8
Fruit					
Frequency	101	0	8	5.7	2.4
Ration	100	0	6	1.9	1.3
Whole Milk					
Frequency	79	0	7	5.1	2.9
Ration	78	0	3	1.0	0.7
0% gm Milk					
Frequency	30	0	7	4.9	3.0
Ration	29	0	2	1.0	0.7
VEGETABLE MILK	1	7	7	7.0	.
Yogurt					
Frequency	101	0	7	3.8	2.8
Ration	101	0	7	0.9	1.0
Whole Cheese					
Frequency	83	0	7	2.4	2.2
Ration	83	0	4	0.90	0.9
0% gm Cheese					
Frequency	32	0	7	1.8	2.4
Ration	32	0	2	0.6	0.7
Red Meat					
Frequency	99	0	7	2.1	1.8
Ration	101	0	4	1.0	0.7
White Meat					
Frequency	102	0	7	2.9	1.6
Ration	102	0	3	1.1	0.6
Fish/Seafood					
Frequency	101	0	7	1.9	1.4
Ration	102	0	3	1.0	0.6
Egg					
Frequency	101	0	7	2.8	1.7
Ration	102	0	4	1.3	0.7
Evoo					
Frequency	94	0	7	5.8	2.2
Ration	88	0	3	1.3	0.8
Sunflower Oil					
Frequency	32	0	7	3.6	3.1
Ration	29	0	4	1.0	1.0
Other oils (ration)	9	0	7	1.6	3.0
Butter					
Frequency	97	0	7	2.1	2.7
Ration	94	0	7	0.7	1.1
Cream					
Frequency	78	0	7	1.3	2.1
Ration	77	0	2	0.4	0.5
Nuts					
Frequency	101	0	7	2.1	2.2
Ration	101	0	3	0.8	0.6
Candy					
Frequency	100	0	7	3.7	2.7
Ration	99	0	5	1.3	1.0
Water					
Frequency	102	0	7	6.7	1.2
Ration	101	0	4.0	1.5	0.7

**Table A2:** Comparison of some demographic, anthropometric and previous obstetrical history data among women with GDM and normoglycemia.

VARIABLE	GDM								*p*-Value
	NO				YES				
	MEAN	SD	MEDIAN	RANK	MEAN	SD	MEDIAN	RANK	
Age	33	5.6	33.65	7	32.22	4.53	33	3	0.67
Residence time in Spain	14.36	9.51	12	30.5	14	1	14	2	0.42
Gestational age	12.09	3.03	12	17	10.63	2.97	11	9	0.19
Number of pregnancies	1.53	0.75	1	3	1.33	0.70	1	2	0.34
Size	162.82	7.34	162	40	161	7.5	160.5	23	0.56
Weight in the first week of gestation	63.78	8.86	63.3	45.7	62.87	12.50	63.5	39	0.86
BMI	24.13	3.38	23.93	20.24	24.40	5.42	22.85	16.24	0.85
Weight prior to gestation	62.78	8.66	62.5	41	62.88	12.78	68	38	0.93
BMI prior to gestation	23.77	3.40	23.01	20.94	23.65	5.20	22.61	15.82	0.78
Previous child weight	3.18	0.57	3.17	2.3	3.03	.	3.03	0	0.89

**Table A3:** Significant differences between women with GDM and normoglycemic women, in the presence of relatives with diabetes, medical history, demographic data.

VARIABLE	*p*-Value
Country of origin	0.55
Marital status	0.36
Work	0.67
Chronic disease	0.47
Allergies	0.05
Relatives with Diabetes	0.021
Previous Diabetes	0.468
Previous GDM	0.43
Insulin treatment in previous GDM	0.28
Previous childbirth	1

**Table A4:** Biochemical results.

PARAMETERS	MEAN
Fasting glycemia	76.70 ± 7.5 (62–104) mg/dL
O’Sullivan 1 h	124.57 ± 29.8 (65–211) mg/dL
OGTT 100 g	79.29 ± 9.124 (57–98) mg/dL
OGTT 100 g 1 h	165.21 ± 28.89 (120–219) mg/dL
OGTT 100 g 2 h	150 ± 40 (84–249) mg/dL
OGTT 100 g 3 h	131.43 ± 32 (89–235) mg/dL
Fasting insulin	10.22 ± 5 (1–18) (U/mL)
HbA1c	5.33 ± 0.388 (4.3–5.7)%
Ferritin	16.86 ± 16.14 (5–89) mg/L
Total plasma protein	6.1 ±0.348 (5.4–6.8) g/dL
Hemoglobin	11.81 ± 1.01 (8.9–14.2) g/dL
Total cholesterol	234 ± 36.40 (174–316) mg/dL
Vitamin D	21 ± 5.55 (12–32) ng/mL

**Table A5:** Perinatal results.

PARAMETERS	MEAN ± SD
GAD	38 ± 4.28 (9–41) weeks
NB weight	3187.82 ± 560.624 (1160–4980) kg
Head circumference	34.119 ± 1.539 (28–37) cm
Apgar 1 min	8.65 ± 0.886 (4–10)
Apgar 5 min	9.61 ± 0.820 (5–10)
Weight percentile	57.081 ± 26.71 (3–99)

**Table A6:** Time of activity during pregnancy in each study group (expressed in minutes).

Variable	DIABETES												*p*-Value
	NO				YES				TOTAL				
	Mean	SD	Median	Rank	Mean	SD	Median	Rank	Mean	SD	Median	Rank	
Time of activity during pregnancy	331.9	227.8	315	780	258.7	188.6	277.5	360	314.71	215.9	315	780	0.684

**Table A7:** Correlation between performing physical activity during pregnancy and the 2 h glucose in the SOG 100 g.

ACTIVITY DURING GESTATION	RHO SPEARMAN	*p-*Value
OGTT 100 g 2 h	−0.426	0.024

**Table A8:** Association between a consumption of white bread with glucose intolerance.

WHITE BREAD	RHO SPEARMAN	*p-*Value
OGTT 2 h	0.450	0.018

**Table A9:** Association between a high consumption of added sugars with glucose intolerance.

ADDED SUGAR	RHO SPEARMAN	*p-*Value
Baseline OGTT	0.396	0.037
OGTT 1 h	0.486	0.009
OGTT 2 h	0.465	0.013
OGTT 3 h	0.434	0.021

**Table A10:** Association between a low consumption of legumes and glucose intolerance in the O’Sullivan test (fasting glucose).

**LEGUME (FC)**	**RHO SPEARMAN**	***p-*Value**
Fasting glycemia	−0.273	0.025
**LEGUME (RATION)**	**RHO SPEARMAN**	***p-*Value**
Fasting glycemia	−0.337	0.005

**Table A11:** Association between lower fish consumption and higher glucose intolerance at baseline O’Sullivan.

FISH (RATION)	RHO SPEARMAN	*p-*Value
Fasting glycemia	−0.294	0.014

**Table A12:** Association between higher consumption of butter and greater glucose intolerance in baseline O’Sullivan.

BUTTER (FC)	RHO SPEARMAN	*p-*Value
Fasting glycemia	0.313	0.010

## References

[1] Plows J.F., Stanley J.L., Baker P.N., Reynolds C.M., Vickers M.H. (2018). The Pathophysiology of Gestational Diabetes Mellitus. Int. J. Mol. Sci..

[2] Mirghani Dirar A., Doupis J. (2017). Gestational diabetes from A to Z. World J. Diabetes.

[3] Zito G., Della Corte L., Giampaolino P., Terzic M., Terzic S., Di Guardo F., Ricci G., Della Pietà I., Maso G., Garzon S. (2020). Gestational diabetes mellitus: Prevention, diagnosis and treatment. A fresh look to a busy corner. J. Neonatal-Perinat. Med..

[4] International Diabetes Federation (IDF) Diabetes Atlas, 10th ed.; 2021; pp. 15–17. Editorial team: Edward J. Boyko, Dianna J. Magliano Suvi Karuranga, Lorenzo Piemonte (et al.). www.diabetesatlas.org.

[5] Metzger B.E., Lowe L.P., Dyer A.R., Trimble E.R., Chaovarindr U., Coustan D.R., Hadden D.R., McCance D.R., Hod M., HAPO Study Cooperative Research Group (2008). Hyperglycemia and Adverse Pregnancy Outcomes. N. Engl. J. Med..

[6] Corcoy R., Lumbreras B., Bartha J.L., Ricart W. (2010). Nuevos criterios diagnósticos de diabetes mellitus gestacional a partir del estudio HAPO. ¿Are they valid in our environment?. Endocrinol. Nutr..

[7] American Diabetes Association (2021). Classification and Diagnosis of Diabetes: Standards of Medical Care in Diabetes—2021. Diabetes Care.

[8] Mousa A., Naqash A., Lim S. (2019). Macronutrient and Micronutrient Intake during Pregnancy: An Overview of Recent Evidence. Nutrients.

[9] Barden A., Singh R., Walters B.N., Ritchie J., Roberman B., Beilin L.J. (2004). Factors predisposing to pre-eclampsia in women with gestational diabetes. J. Hypertens..

[10] Zhang X., Liao Q., Wang F., Li D. (2018). Association of gestational diabetes mellitus and abnormal vaginal flora with adverse pregnancy outcomes. Medicine.

[11] Pallardo Sánchez L.F., Bartha Rasero J.L., Herranz de la Morena L. (2015). Diabetes y Embarazo.

[12] Zhang R., Han S., Chen G.-C., Li Z.-N., Silva-Zolezzi I., Parés G.V., Wang Y., Qin L.-Q. (2018). Effects of low-glycemic-index diets in pregnancy on maternal and newborn outcomes in pregnant women: A meta-analysis of randomized controlled trials. Eur. J. Nutr..

[13] Fugmann M., Breier M., Rottenkolber M., Banning F., Ferrari U., Sacco V., Grallert H., Parhofer K.G., Seissler J., Clavel T. (2015). The stool microbiota of insulin resistant women with recent gestational diabetes, a high risk group for type 2 diabetes. Sci. Rep..

[14] Jayashree B., Bibin Y.S., Prabhu D., Shanthirani C.S., Gokulakrishnan K., Lakshmi B.S., Mohan V., Balasubramanyam M. (2014). Increased circulatory levels of lipopolysaccharide (LPS) and zonulin signify novel biomarkers of proinflammation in patients with type 2 diabetes. Mol. Cell. Biochem..

[15] Gomez-Arango L.F., Barrett H.L., McIntyre H.D., Callaway L.K., Morrison M., Nitert M.D. (2016). Connections Between the Gut Microbiome and Metabolic Hormones in Early Pregnancy in Overweight and Obese Women. Diabetes.

[16] David L.A., Maurice C.F., Carmody R.N., Gootenberg D.B., Button J.E., Wolfe B.E., Ling A.V., Devlin A.S., Varma Y., Fischbach M.A. (2014). Diet rapidly and reproducibly alters the human gut microbiome. Nature.

[17] Bo S., Lezo A., Menato G., Gallo M.-L., Bardelli C., Signorile A., Berutti C., Massobrio M., Pagano G.F. (2005). Gestational hyperglycemia, zinc, selenium, and antioxidant vitamins. Nutrition.

[18] Debreceni B., Debreceni L. (2014). The Role of Homocysteine-Lowering B-Vitamins in the Primary Prevention of Cardiovascular Disease. Cardiovasc. Ther..

[19] Patterson S., Flatt P., Brennan L., Newsholme P., McClenaghan N.H. (2006). Detrimental actions of metabolic syndrome risk factor, homocysteine, on pancreatic β-cell glucose metabolism and insulin secretion. J. Endocrinol..

[20] Gong T., Wang J., Yang M., Shao Y., Liu J., Wu Q., Xu Q., Wang H., He X., Chen Y. (2016). Serum homocysteine level and gestational diabetes mellitus: A meta-analysis. J. Diabetes Investig..

[21] Zhang C., Williams M.A., Sorensen T.K., King I.B., Kestin M.M., Thompson M.L., Leisenring W.M., Dashow E.E., Luthy D.A. (2004). Maternal Plasma Ascorbic Acid (Vitamin C) and Risk of Gestational Diabetes Mellitus. Epidemiology.

[22] Amraei M., Mohamadpour S., Sayehmiri K., Mousavi S.F., Shirzadpour E., Moayeri A. (2018). Effects of Vitamin D Deficiency on Incidence Risk of Gestational Diabetes Mellitus: A Systematic Review and Meta-analysis. Front. Endocrinol..

[23] Zhang C., Qiu C., Hu F.B., David R.M., van Dam R., Bralley A., Williams M.A. (2008). Maternal Plasma 25-Hydroxyvitamin D Concentrations and the Risk for Gestational Diabetes Mellitus. PLoS ONE.

[24] Dolatkhah N., Hajifaraji M., Shakouri S.K. (2018). Nutrition Therapy in Managing Pregnant Women with Gestational Diabetes Mellitus: A Literature Review. J. Fam. Reprod. Health.

[25] Allehdan S., Basha A., Tayyem R. (2020). Gestational diabetes mellitus management: Diet and lifestyle. Nutr. Food Sci..

[26] Walsh J.M., McGowan C.A., Mahony R., Foley M.E., McAuliffe F.M. (2012). Low glycaemic index diet in pregnancy to prevent macrosomia (ROLO study): Randomised control trial. BMJ.

[27] Moreno-Castilla C., Hernandez M., Bergua M., Alvarez M.C., Arce M.A., Rodriguez K., Martinez-Alonso M., Iglesias M., Mateu M., Santos M.D. (2013). Low-Carbohydrate Diet for the Treatment of Gestational Diabetes Mellitus: A randomized controlled trial. Diabetes Care.

[28] Cremona A., O’Gorman C., Cotter A., Saunders J., Donnelly A. (2018). Effect of exercise modality on markers of insulin sensitivity and blood glucose control in pregnancies complicated with gestational diabetes mellitus: A systematic review. Obes. Sci. Pract..

[29] Thorens B. (2008). Glucose sensing and the pathogenesis of obesity and type 2 diabetes. Int. J. Obes..

[30] Cai S., Tan S., Gluckman P.D., Godfrey K.M., Saw S.-M., Teoh O.H., Chong Y.-S., Meaney M.J., Kramer M.S., Gooley J.J. (2017). Sleep Quality and Nocturnal Sleep Duration in Pregnancy and Risk of Gestational Diabetes Mellitus. Sleep.

[31] Salavati N., Vinke P.C., Lewis F., Bakker M.K., Erwich J.J.H., Der Beek E.M.M. (2020). Offspring Birth Weight is Associated with Specific Preconception Maternal Food Group Intake: Data from a Linked Population-Based Birth Cohort. Nutrients.

