# Supplementary material for: Influence of Diet and Lifestyle on the Development of Gestational Diabetes Mellitus and on Perinatal Results
Source: Nutrients. 2022 Jul 19;14(14):2954. doi: 10.3390/nu14142954 (PMC9319526; doi:10.3390/nu14142954)
Supplement: Supplementary file 1 [file nutrients-14-02954-s001.zip › nutrients-1805860-supplementary.pdf]

Hospital Universitario La Paz
SaludMadrid
Comunidad de Madrid

CEU Universidad San Pablo

OCTUBRE DE 2018

CUESTIONARIO PARA GESTANTES

NUTRICIÓN

En cumplimiento de lo dispuesto en la ley 15/1999, de 13 de diciembre, de Protección de Datos Personales (LOPD), le informamos que los datos personales obtenidos para este estudio son confidenciales y serán tratados de conformidad con lo establecido en la legislación vigente. Sólo tendrán acceso a los mismos los investigadores del estudio y para los fines que se le han indicado anteriormente. En cualquier momento usted podrá ejercitar los derechos de acceso, rectificación, oposición y, en su caso, cancelación, comunicándolo por escrito con indicación de sus datos a IDiPAZ- Dr. José Luis Bartha, en el domicilio Paseo Castellana 261, 28046 de Madrid.

CUESTIONARIO DE NUTRICION PARA GESTANTES

Mediante el siguiente cuestionario se va a valorar como la dieta y hábitos de vida de la gestante pueden influir posteriormente en el desarrollo de patologías como puede ser la Diabetes gestacional.

I. DATOS IDENTIFICATIVOS:

Nombre y apellidos:

Domicilio:

Provincia:

Teléfono:

País de origen:

Tiempo de residencia en España:

Área de Salud:

II. DATOS DEMOGRÁFICOS (Rodee la opción correcta)

Estado civil: Soltera, Casada, Divorciada, Viuda

Trabajo: SI, NO.

Especifique cual y que puesto desempeña…………………………………………….......

Nivel de estudios: ……………………………………………………………………

III. DATOS ANTROPOMÉTRICOS

1. Número de semanas de gestación……………………………………………………...
2. Número de hijos totales contando con éste……………………………………………
3. Planifico su embarazo
    a) Si
    b) No
4. ¿Cuánto mide usted?...
5. ¿Cuál es su peso actual?..
6. ¿Cuál era su preso previo a la gestación?..
7. De acuerdo con su peso previo, puede decir que era:
    a) Delgada
    b) Peso adecuado
    c) Sobrepeso
    d) Obesa
8. ¿Cuántos kilos cree que debería ganar <u>en total</u> a lo largo de todo el embarazo?...Kg
9. ¿Tiene conocimiento de cómo puede afectar a su salud y a la de su bebe una ganancia de peso excesiva?
    a) Si
    b) No

IV. HISTORIA CLÍNICA

10. ¿Padece alguna enfermedad crónica?
    a) Si
    b) No

    Indique cual: ………………………………………………………………......
11. ¿Tiene alergias? (Fármacos, alimentos, intolerancias alimentarias)

    Indique cuales……………………………………………………………………
12. ¿Tiene familiares directos con diabetes o hipertensión?
    a) Si
    b) No

    Indique quien: …………………………………………………………………
13. ¿Ha tenido problemas de glucosa o presión arterial previos al embarazo?
    a) Si
    b) No

14. ¿En embarazos previos tuvo diabetes gestacional o problemas de intolerancia a la glucosa?
    a) Si
    b) No

**En caso de haber padecido Diabetes Gestacional en embarazos previos:**
15. ¿Pudo controlar sus glucemias mediante dieta y ejercicio físico?<br>a) Si<br>b) No<br>16. ¿Precisó tratamiento con insulina?<br>a) Si<br>b) No<br>17. En caso de tener conocimiento de ello, indique que tipo de insulina tuvo durante el tratamiento:<br>a) Insulina de acción intermedia NPH<br>b) Insulina de acción Lenta<br>c) Insulina de acción Rápida<br>d) Tratamiento combinado de Insulina de acción Lenta y Rápida<br>e) Insulina tipo (Mezcla)<br>18. En caso de tener conocimiento de ello. Indique que nivel de hemoglobina glicosilada (HbA1c) tuvo durante la gestación o en su último control: ……………………………………………………………………………..<br>19. ¿Controlaba sus niveles de glucosa mediante la realización de glucemias pre y postprandiales?<br>a) Si<br>b) No

20. ¿Cuánto peso su anterior hijo al nacer?...
21. ¿Su anterior parto fue natural o cesarea?..

V. HABITOS ALIMENTICIOS

22. ¿Siente más apetito ahora al estar embarazada?
    a) Si
    b) No

23. ¿Hay algunos alimentos que le gusten más ahora al estar embarazada?

a) Si

b) No

Especifique cuales…………………………………………………………………….

24. ¿Hay algunos alimentos que no le gusten ahora al estar embarazada o que haya disminuido su consumo?

a) Si

b) No

Especifique cuales…………………………………………………………………….

25. ¿Hay algunos alimentos que le sienten mal ahora al estar embarazada?

a) Si

b) No

Especifique cuales…………………………………………………………………….

26. ¿Está teniendo antojos?

a) Si

b) No

Especifique los alimentos antojados…………………………………………………...

27. ¿Sigue algún tipo de régimen?

a) No, ninguno

b) Si, control de peso

c) Si, hipertensión

d) Si, diabetes

e) Si, colesterol

f) Otro, especificar……………………………………………………………………

28. ¿Quién le ha indicado la necesidad de realizar régimen?

a) Iniciativa propia

b) Médico

c) Matrona/ Enfermera

d) Otro, especificar…………………………………………………………………...

29. ¿Cuántas veces come al día de forma habitual?..

30. ¿Cuál de estas comidas realiza habitualmente?
    a) Desayuno
    b) Media mañana
    c) Comida
    d) Merienda
    e) Cena
    f) Media noche
    g) Otros

31. En la siguiente tabla indique la frecuencia con la que suele consumir los siguientes alimentos, en **veces por semana** y la ración aproximada de ellos.

ALIMENTOS	FRECUENCIA DE CONSUMO	CANTIDAD O RACIONES
Pan blanco 1 Ración=20 gr<br>Pan integral		
Pasta, arroz, otros cereales		
Legumbres		
Verdura u hortalizas<br>1 ración= 200gr (1 plato)		
Fruta		
Leche (especificar si es 0% MG)		
Yogures o lácteos fermentados		
Quesos (especificar si es 0% MG)		
Carnes rojas y vísceras: ternera, hamburguesas, salchichas, jamón, embutidos.<br>(1 ración/pieza= 100gr)		
Carnes blancas: pollo, pavo, conejo…		

Pescados o mariscos (1 pieza= 100gr de pescado)		
Huevos		
Aceite (especificar si oliva, girasol, coco…)		
Mantequilla, margarina, nata		
Frutos secos		
Dulces: galletas, flanes, pasteles		
Agua		
Bebidas con azúcar (zumos, refrescos…)		
Añade azúcar a las bebidas (café, té)		
Bebidas alcohólicas		

VI. ESTILO DE VIDA

32. ¿Fumaba antes de la gestación?
    a) Si
    b) No
33. En caso afirmativo, ¿Ha dejado de fumar con motivo del embarazo?
    a) Si
    b) No
34. Número de cigarrillos al día………………………………………………………......
35. ¿Consume bebidas con alcohol?
    a) Si
    b) No

    Indique tipo de bebida, frecuencia y cantidad de consumo……………………………

36. Consume bebidas con cafeína (café, té, refrescos de cola…)
    a) Si
    b) No

    Indique tipo de bebida, frecuencia y cantidad de consumo……………………………

37. ¿Consume alimentos ultra procesados, dulces, alimentos con grasas saturadas, azucares añadidos?
    a) Si
    b) No

    Indique tipo de alimento y frecuencia de consumo…………………………………….

38. ¿Realizaba actividad física aeróbica o anaeróbica antes de la gestación?
    a) Si
    b) No, llevaba una vida sedentaria

    Indique tipo de ejercicio y tiempo de realización diaria/semanal…………………….

39. ¿En la actualidad durante la gestación está realizando algún tipo de ejercicio físico?
    a) Si
    b) No

    Indique tipo de ejercicio y tiempo de realización diaria/semanal……………………...

VII. CONSUMO DE SUPLEMENTOS

40. ¿Ha comenzado a tomar algún tipo de suplemento para el embarazo?
    a) Si
    b) No

En caso afirmativo indique el tipo de suplemento, la razón y quien se lo indicó.

TIPO DE SUPLEMENTO	RAZÓN<br>Preventivo, deficiencia, otras	INDICADO POR<br>(Medico, iniciativa propia, otros)
Hierro		
Calcio		
Vitaminas (A,D,E,B2,B6,B9,C…)		
Otros (especificar)		

41. ¿Ha tomado algún tipo de suplemento en los meses previos a la gestación?
   a) Si
   b) No

   En caso afirmativo indique el tipo de suplemento, la razón y quien se lo indicó.

TIPO DE SUPLEMENTO	CUANDO LO TOMO (Meses antes del embarazo)	RAZÓN (Preventivo, deficiencia, otras)	INDICADO POR (Medico, iniciativa propia, otros)
Hierro			
Calcio			
Vitaminas (A,D,E,B2,B6,B9,C…)			
Otros (especificar)			

VIII. CONOCIMIENTOS

42. ¿Qué ganancia de peso es la aconsejada en total en el embarazo?
   a) De 12 a 20 kg
   b) De 9 a 12,5 kg
   c) De 6 a 9 kg
43. Indique cuales de los siguientes alimentos es conveniente consumir con moderación para prevenir el exceso de grasa/colesterol en la sangre. (Tache la opción que considere correcta)

   Aceite de oliva ☐ Quesos curados ☐
   Huevos ☐ Cremas y salsas ☐
   Pan ☐ Salmón ☐
   Chorizo☐ Bollería ☐
   Margarina ☐ Frutos secos ☐
   Aguacate ☐ Cacao puro al 80% ☐
44. Indique que alimentos es conveniente consumir con moderación por su alto contenido en azucares.

   Zumos de frutas comerciales ☐ Legumbres ☐
   Miel ☐ Frutas☐
   Pasta ☐ Pan ☐

45. Indique cuantos minutos de actividad diaria moderada son necesarios para mantener la salud cardiovascular/materno-fetal.
    a) 15 min
    b) 30-60 min
46. En cuanto al calcio; indique la respuesta correcta
    a) Es esencial para el metabolismo del calcio y fosforo
    b) Durante el embarazado se produce una transferencia de calcio de la madre al feto
    c) En ocasiones se aconseja la administración de suplementos de 400 UN/día para cubrir las necesidades del feto
    d) Todas son correctas
47. Indique cuales de los siguientes alimentos son ricos en calcio

Brócoli □	Leche □
Almendras□	Tomate □
Sardinas□	

48. Indique cuál de los siguientes alimentos son una buena fuente de vitamina C
    a) Kiwi
    b) Aceite de oliva
    c) Plátano
49. En cuanto al ácido Fólico/ vitamina B9: Indique la respuesta correcta
    a) Sus niveles en sangre disminuyen durante la gestación debido a las demandas fetales
    b) Entre otros problemas, una deficiencia de folatos podrá desencadenar anomalías en el sistema nervioso del feto; alteraciones en el desarrollo del tubo neural.
    c) La ingesta recomendada puede ser el **doble de una mujer adulta no gestante** aproximadamente 400 microgramos/día.
    d) Todas son correctas
50. Indique cuál de los siguientes alimentos son una buena fuente de ácido fólico/ vitamina B9.
    a) Espinacas
    b) Manzanas
    c) Arroz

51. ¿Cree que durante la gestación debe comer por dos?

    a) Si
    b) No, pero si es cierto que las necesidades nutricionales aumentan.

52. ¿La dieta y estilo de vida de la gestante pueden influir en el desarrollo de enfermedades tanto maternas como fetales?

    a) Si
    b) No

53. ¿Cree que la alimentación durante el embarazo puede repercutir en la salud futura de su hijo?

    a) Si
    b) No

54. ¿Cree que su alimentación y estilo de vida son correctos?

    a) No
    b) Poco
    c) Bastante, pero podría mejorar
    d) Mucho

55. Responda verdadero (V), falso (F) a las siguientes afirmaciones.

    a. El aceite de oliva virgen extra es una grasa saludable…………………………...
    b. La margarina tiene menos inconvenientes para la salud que la mantequilla……….
    c. La carencia de ácido fólico en las primeras semanas de gestación puede producir malformaciones del tubo neural fetal…………………………………………...
    d. El hierro de los alimentos se absorbe mejor combinándolo con alimentos ricos en vitamina C…………………………………………………………………………
    e. La luz solar es precursora de la vitamina D, necesaria para la fijación del Calcio en nuestro organismo…………………………………………………………...
    f. El sedentarismo y sobrepeso son factores de riesgo para el desarrollo de Diabetes gestacional…………………………………………………………………...

56. ¿Le gustaría recibir más información nutricional durante la gestación que le ayudara así a prevenir patologías gestacionales?

    a) Si
    b) No, la que tengo es suficiente
